# Ethyl *N*-(3-cyano-1*H*-indol-2-yl)form­imidate

**DOI:** 10.1107/S1600536813029589

**Published:** 2013-11-06

**Authors:** Yang Ruchun, Zhang Hui, Cao BanPeng

**Affiliations:** a Jiangxi Key Laboratory of Organic Chemistry, Jiangxi Science & Technology Normal University, Nanchang 330013, People’s Republic of China

## Abstract

In the title compound, C_12_H_11_N_3_O, the C=N imino bond is in an *E* conformation. In the crystal, adjacent mol­ecules are linked by N–H⋯N_cyano_ hydrogen bonds, forming a chain running along [110].

## Related literature

The starting reactant was synthesized according to a literature method (Yang *et al.*, 2010 ▶). Introduction of different groups into indole mol­ecules can generate a series of bioactive derivatives, which have been the subject of much attention as anti-cancer drugs (Laird *et al.*, 2000 ▶; Li *et al.*, 2005 ▶).
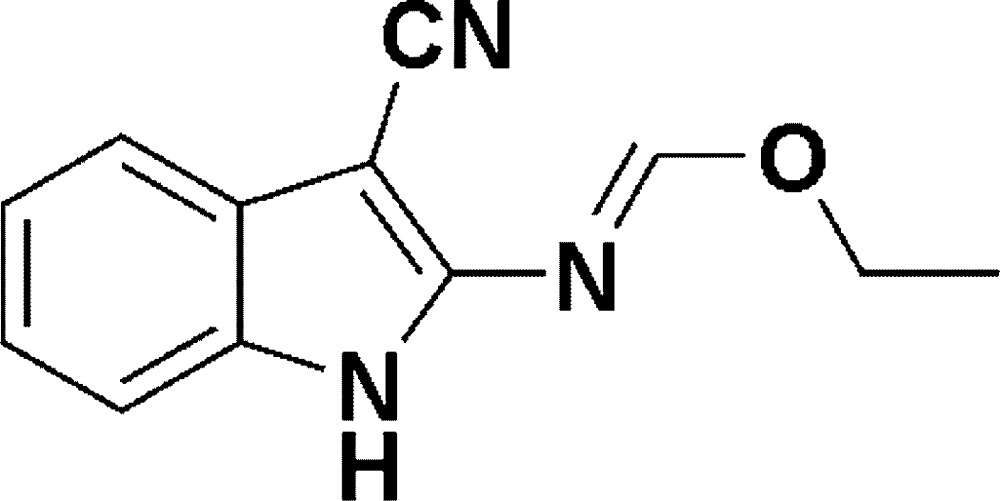


## Experimental

### 

#### Crystal data

C_12_H_11_N_3_O
*M* *_r_* = 213.24
Monoclinic, 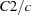

*a* = 12.7884 (6) Å
*b* = 8.0546 (6) Å
*c* = 21.4116 (10) Å
β = 94.069 (4)°
*V* = 2200.0 (2) Å^3^
*Z* = 8
Mo *K*α radiation
μ = 0.09 mm^−1^
*T* = 296 K
0.30 × 0.20 × 0.20 mm

#### Data collection

Bruker SMART APEX diffractometer
Absorption correction: multi-scan (*SADABS*; Sheldrick, 1996 ▶) *T*_min_ = 0.997, *T*_max_ = 0.998
14358 measured reflections
1934 independent reflections
1579 reflections with *I* > 2σ(*I*)
*R*_int_ = 0.031

#### Refinement

*R*[*F* ^2^ > 2σ(*F* ^2^)] = 0.040
*wR*(*F* ^2^) = 0.110
*S* = 1.12
1934 reflections
149 parameters
379 restraints
H atoms treated by a mixture of independent and constrained refinement
Δρ_max_ = 0.18 e Å^−3^
Δρ_min_ = −0.19 e Å^−3^

### 

Data collection: *SMART* (Bruker, 1997 ▶); cell refinement: *SAINT* (Bruker, 1997 ▶); data reduction: *SAINT*; program(s) used to solve structure: *SHELXS97* (Sheldrick, 2008 ▶); program(s) used to refine structure: *SHELXL97* (Sheldrick, 2008 ▶); molecular graphics: *XP* in *SHELXTL* (Sheldrick, 2008 ▶); software used to prepare material for publication: *SHELXTL*.

## Supplementary Material

Crystal structure: contains datablock(s) I, global. DOI: 10.1107/S1600536813029589/ng5344sup1.cif

Structure factors: contains datablock(s) I. DOI: 10.1107/S1600536813029589/ng5344Isup2.hkl

Additional supplementary materials:  crystallographic information; 3D view; checkCIF report

## Figures and Tables

**Table 1:** Hydrogen-bond geometry (Å, °)

*D*—H⋯*A*	*D*—H	H⋯*A*	*D*⋯*A*	*D*—H⋯*A*
N1—H1⋯N2^i^	0.87 (2)	2.12 (2)	2.9490 (19)	161 (2)

Symmetry code: (i) 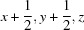.

**Fig. 1.**
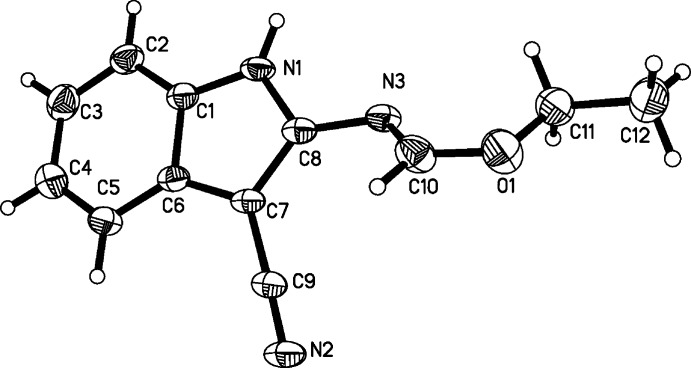
Thermal ellipsoid plot of C12H11N3O. Ellipsoids are drawn at the 30% probability level and H atoms are represented as small spheres of arbitrary radius.

**Fig. 2.**
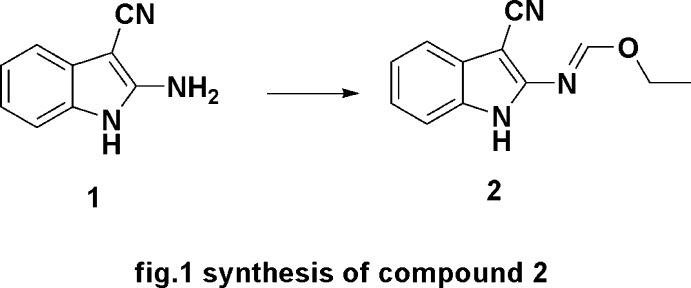
Synthesis method of the title compound.

**Fig. 3.**
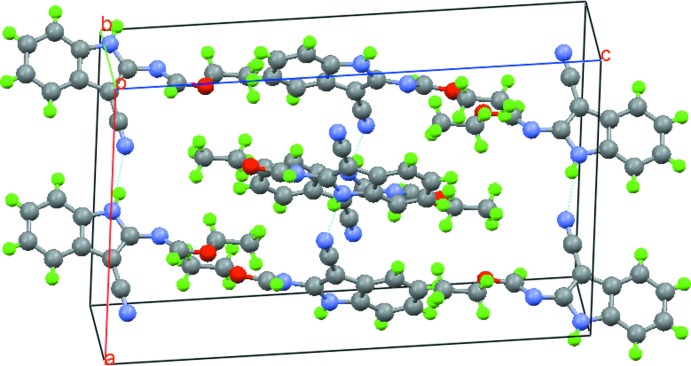
The packing of the title compound, viewed down the c axis. Dashed lines indicate hydrogen bonds.

## supplementary crystallographic information

### 1. Comment

The indole compounds play an important role in the pharmaceutical and agrochemical industries. Introduction of different groups into indole molecules can generate a series of bioactive derivatives, which have been the subject of much attention in anti-cancer drugs (Laird *et al.*, 2000; Li *et al.*, 2005). In our study, we report an indole compound.

### 2. Experimental

The starting reactant 1 was synthesized according to a literature method (Yang *et al.*, 2010).

Synthesis of (2)

2-amino-1*H*-indole-3-carbonitrile (6.29 g, 40 mmol) was suspended in dry acetonitrile (150 ml). Triethylorthoformate (10.92 ml, 9.73 g, 60 mmol) was added and the mixture was heated at reflux temperature for 1 hour. The dark brown solution was cooled to room temperature and filtered through filter paper. The acetonitrile was removed on a rotoevaporator to afford 2 as a brown solid. ^1^H NMR (400 MHz, CDCl_3_, TMS): δ 1.41(t, 3H, *J*=7.2 Hz, OCH_2_CH_3_), 4.40 (q, 2H, *J*=7.2 Hz, OCH_2_CH_3_), 7.20-7.31 (m, 3H, aromatic H), 7.61 (dd, 1H, aromatic H), 8.51 (s, 1H, N=CH) ppm; ^13^C NMR (100 MHz, CDCl_3_): δ 14.0, 64.0, 72.7, 111.0, 116.7, 118.9, 122.2, 123.2, 127.5, 132.2, 149.8, 160.7 ppm. Crystals were grown from an acetonitrile solution.

### 3. Refinement

H atoms bond to N were located in a difference map and refined with distance of N—H = 0.866 Å (18) and *U*_iso_(H) = 1.2*U*_eq_(N). other H atoms attached to C were fixed geometrically and treated as riding with C—H = 0.96 Å (methyl) or 0.93 Å (aromatic) and with *U*_iso_(H) = 1.2*U*_eq_(aromatic) or *U*_iso_(H) = 1.5*U*_eq_(methyl).

### Crystal data

**Table:**

C_12_H_11_N_3_O	*F*(000) = 896
*M**_r_* = 213.24	*D*_x_ = 1.288 Mg m^−^^3^
Monoclinic, *C*2/*c*	Mo *K*α radiation, λ = 0.71073 Å
Hall symbol: -C 2yc	Cell parameters from 1934 reflections
*a* = 12.7884 (6) Å	θ = 3.0–26.2°
*b* = 8.0546 (6) Å	µ = 0.09 mm^−^^1^
*c* = 21.4116 (10) Å	*T* = 296 K
β = 94.069 (4)°	Block, colorless
*V* = 2200.0 (2) Å^3^	0.30 × 0.20 × 0.20 mm
*Z* = 8	

### Data collection

**Table:**

Bruker SMART APEX diffractometer	1934 independent reflections
Radiation source: fine-focus sealed tube	1579 reflections with *I* > 2σ(*I*)
Graphite monochromator	*R*_int_ = 0.031
ω scans	θ_max_ = 25.0°, θ_min_ = 3.0°
Absorption correction: multi-scan (*SADABS*; Sheldrick, 1996)	*h* = −15→15
*T*_min_ = 0.997, *T*_max_ = 0.998	*k* = −9→9
14358 measured reflections	*l* = −25→25

### Refinement

**Table:**

Refinement on *F*^2^	Primary atom site location: structure-invariant direct methods
Least-squares matrix: full	Secondary atom site location: difference Fourier map
*R*[*F*^2^ > 2σ(*F*^2^)] = 0.040	Hydrogen site location: inferred from neighbouring sites
*wR*(*F*^2^) = 0.110	H atoms treated by a mixture of independent and constrained refinement
*S* = 1.12	*w* = 1/[σ^2^(*F*_o_^2^) + (0.0524*P*)^2^ + 0.9005*P*] where *P* = (*F*_o_^2^ + 2*F*_c_^2^)/3
1934 reflections	(Δ/σ)_max_ < 0.001
149 parameters	Δρ_max_ = 0.18 e Å^−^^3^
379 restraints	Δρ_min_ = −0.19 e Å^−^^3^

### Special details

**Table:**

Geometry. All esds (except the esd in the dihedral angle between two l.s. planes) are estimated using the full covariance matrix. The cell esds are taken into account individually in the estimation of esds in distances, angles and torsion angles; correlations between esds in cell parameters are only used when they are defined by crystal symmetry. An approximate (isotropic) treatment of cell esds is used for estimating esds involving l.s. planes.
Refinement. Refinement of F^2^ against ALL reflections. The weighted R-factor wR and goodness of fit S are based on F^2^, conventional R-factors R are based on F, with F set to zero for negative F^2^. The threshold expression of F^2^ > 2sigma(F^2^) is used only for calculating R-factors(gt) etc. and is not relevant to the choice of reflections for refinement. R-factors based on F^2^ are statistically about twice as large as those based on F, and R- factors based on ALL data will be even larger.

### Fractional atomic coordinates and isotropic or equivalent isotropic displacement parameters (Å^2^)

**Table:**

	*x*	*y*	*z*	*U*_iso_*/*U*_eq_	
N1	0.49318 (10)	0.29181 (16)	0.48715 (6)	0.0443 (3)	
H1	0.5435 (14)	0.348 (2)	0.4722 (8)	0.053*	
N2	0.19409 (11)	−0.05378 (19)	0.46004 (7)	0.0594 (4)	
N3	0.40141 (10)	0.25137 (15)	0.38870 (6)	0.0452 (3)	
O1	0.34656 (11)	0.16502 (16)	0.29081 (6)	0.0700 (4)	
C1	0.48809 (11)	0.25080 (18)	0.54931 (7)	0.0421 (4)	
C2	0.54626 (13)	0.3073 (2)	0.60195 (8)	0.0529 (4)	
H2	0.6022	0.3797	0.5987	0.063*	
C3	0.51832 (15)	0.2523 (2)	0.65929 (9)	0.0618 (5)	
H3	0.5557	0.2893	0.6955	0.074*	
C4	0.43506 (15)	0.1423 (2)	0.66423 (8)	0.0618 (5)	
H4	0.4186	0.1061	0.7036	0.074*	
C5	0.37706 (13)	0.0865 (2)	0.61200 (8)	0.0525 (4)	
H5	0.3214	0.0137	0.6157	0.063*	
C6	0.40314 (11)	0.14121 (17)	0.55334 (7)	0.0396 (4)	
C7	0.35841 (10)	0.11823 (17)	0.49076 (7)	0.0393 (4)	
C8	0.41570 (11)	0.21415 (17)	0.45158 (7)	0.0401 (4)	
C9	0.26740 (12)	0.02327 (19)	0.47287 (7)	0.0436 (4)	
C10	0.36983 (15)	0.1409 (2)	0.35136 (8)	0.0589 (5)	
H10	0.3621	0.0343	0.3670	0.071*	
C11	0.35402 (15)	0.3329 (2)	0.26845 (8)	0.0627 (5)	
H11A	0.4222	0.3788	0.2814	0.075*	
H11B	0.3008	0.4014	0.2857	0.075*	
C12	0.33881 (17)	0.3308 (3)	0.19911 (9)	0.0765 (6)	
H12A	0.3929	0.2653	0.1823	0.115*	
H12B	0.3420	0.4423	0.1834	0.115*	
H12C	0.2716	0.2836	0.1867	0.115*	

### Atomic displacement parameters (Å^2^)

**Table:**

	*U*^11^	*U*^22^	*U*^33^	*U*^12^	*U*^13^	*U*^23^
N1	0.0337 (7)	0.0414 (7)	0.0582 (8)	−0.0134 (6)	0.0058 (6)	0.0020 (6)
N2	0.0426 (8)	0.0631 (9)	0.0725 (10)	−0.0232 (7)	0.0051 (6)	0.0006 (7)
N3	0.0391 (7)	0.0447 (7)	0.0523 (8)	−0.0114 (6)	0.0070 (5)	−0.0010 (5)
O1	0.0946 (10)	0.0603 (8)	0.0553 (8)	−0.0194 (7)	0.0057 (7)	−0.0082 (6)
C1	0.0338 (8)	0.0366 (8)	0.0557 (9)	−0.0016 (6)	0.0025 (6)	0.0021 (6)
C2	0.0446 (9)	0.0448 (9)	0.0678 (11)	−0.0089 (8)	−0.0064 (8)	0.0016 (8)
C3	0.0642 (11)	0.0599 (11)	0.0592 (11)	−0.0026 (9)	−0.0107 (8)	−0.0003 (8)
C4	0.0637 (11)	0.0688 (12)	0.0527 (10)	−0.0039 (10)	0.0037 (8)	0.0083 (9)
C5	0.0443 (9)	0.0529 (10)	0.0611 (10)	−0.0072 (8)	0.0083 (7)	0.0083 (8)
C6	0.0300 (7)	0.0342 (7)	0.0547 (9)	−0.0005 (6)	0.0047 (6)	0.0010 (6)
C7	0.0284 (7)	0.0349 (7)	0.0551 (9)	−0.0055 (6)	0.0069 (6)	−0.0008 (6)
C8	0.0322 (7)	0.0337 (7)	0.0548 (9)	−0.0039 (6)	0.0068 (6)	−0.0021 (6)
C9	0.0354 (8)	0.0412 (8)	0.0550 (9)	−0.0062 (7)	0.0087 (6)	0.0015 (7)
C10	0.0729 (12)	0.0487 (9)	0.0563 (11)	−0.0157 (8)	0.0117 (8)	−0.0050 (7)
C11	0.0639 (11)	0.0637 (12)	0.0609 (11)	−0.0036 (9)	0.0071 (8)	0.0010 (8)
C12	0.0734 (13)	0.0910 (15)	0.0636 (12)	0.0029 (12)	−0.0056 (10)	0.0011 (10)

### Geometric parameters (Å, º)

**Table:**

N1—C8	1.3587 (19)	C4—C5	1.373 (2)
N1—C1	1.377 (2)	C4—H4	0.9300
N1—H1	0.866 (18)	C5—C6	1.393 (2)
N2—C9	1.1415 (19)	C5—H5	0.9300
N3—C10	1.244 (2)	C6—C7	1.431 (2)
N3—C8	1.379 (2)	C7—C8	1.387 (2)
O1—C10	1.324 (2)	C7—C9	1.422 (2)
O1—C11	1.440 (2)	C10—H10	0.9300
C1—C2	1.383 (2)	C11—C12	1.484 (3)
C1—C6	1.407 (2)	C11—H11A	0.9700
C2—C3	1.376 (2)	C11—H11B	0.9700
C2—H2	0.9300	C12—H12A	0.9600
C3—C4	1.395 (3)	C12—H12B	0.9600
C3—H3	0.9300	C12—H12C	0.9600
			
C8—N1—C1	110.41 (12)	C8—C7—C9	126.43 (14)
C8—N1—H1	124.4 (11)	C8—C7—C6	107.52 (12)
C1—N1—H1	124.7 (11)	C9—C7—C6	125.92 (13)
C10—N3—C8	119.12 (14)	N1—C8—N3	119.22 (13)
C10—O1—C11	116.60 (14)	N1—C8—C7	108.23 (13)
N1—C1—C2	130.48 (14)	N3—C8—C7	132.25 (13)
N1—C1—C6	107.42 (13)	N2—C9—C7	178.31 (16)
C2—C1—C6	121.97 (15)	N3—C10—O1	124.39 (16)
C3—C2—C1	117.56 (16)	N3—C10—H10	117.8
C3—C2—H2	121.2	O1—C10—H10	117.8
C1—C2—H2	121.2	O1—C11—C12	108.37 (16)
C2—C3—C4	121.33 (16)	O1—C11—H11A	110.0
C2—C3—H3	119.3	C12—C11—H11A	110.0
C4—C3—H3	119.3	O1—C11—H11B	110.0
C5—C4—C3	121.14 (16)	C12—C11—H11B	110.0
C5—C4—H4	119.4	H11A—C11—H11B	108.4
C3—C4—H4	119.4	C11—C12—H12A	109.5
C4—C5—C6	118.74 (15)	C11—C12—H12B	109.5
C4—C5—H5	120.6	H12A—C12—H12B	109.5
C6—C5—H5	120.6	C11—C12—H12C	109.5
C5—C6—C1	119.25 (14)	H12A—C12—H12C	109.5
C5—C6—C7	134.24 (14)	H12B—C12—H12C	109.5
C1—C6—C7	106.42 (13)		

### Hydrogen-bond geometry (Å, º)

**Table:**

*D*—H···*A*	*D*—H	H···*A*	*D*···*A*	*D*—H···*A*
N1—H1···N2^i^	0.87 (2)	2.12 (2)	2.9490 (19)	161 (2)

Symmetry code: (i) *x*+1/2, *y*+1/2, *z*.
